# Overcoming gaps in the treatment of neurodegenerative disease

**DOI:** 10.1016/j.ebiom.2020.103088

**Published:** 2020-10-19

As health-care systems have adjusted to try and deal with the unprecedented scale of the COVID-19 pandemic, many routine health services were severely disrupted and gaps across social care settings have become increasingly apparent. These challenges were particularly evident when looking at the support of those with neurodegenerative diseases. An international report led by University College London (London, UK) examined the impact of COVID-19 on people with dementia and estimated mortality rates of up to 75%. In the same time period, The Alzheimer's Society (London, UK) expressed concern about a 4% decline in dementia diagnosis rates.

Even before the COVID-19 pandemic the high rates of people with undiagnosed dementia has been an ongoing concern. The global prevalence of undiagnosed dementia is higher than 60%, according to a review published by the British Medical Journal in 2017. The analysis, led by Ruoling Chen (University of Wolverhampton, UK), highlighted wide variations in dementia detection among different countries, socioeconomic backgrounds, and in communities, compared with residential nursing settings.

Although there are no available treatments to cure neurodegenerative conditions, such as Parkinson's or Alzheimer's disease, there is an increasing range of therapeutic and supportive options that are available. Early diagnosis is essential for treatment planning and to ensure that the right support can be provided to patients and their families.

In February 2020, The Royal Society held a conference on healthy ageing as part of their Transforming our Future series. Researchers discussed advances across research and social care that are needed to support the ambitious Grand Challenge announced by the UK Government to achieve 5 extra years of health in old age by 2035. With age being the biggest risk factor in neurodegenerative disease, many of the talks were focused on elucidating pathological mechanisms and developing new interventions for these conditions. Payam Barnaghi (University of Surrey, UK) described his work to combine robotics, artificial intelligence, and big data to create and test so-called healthy home technology that would support independent living for those with dementia. The approach uses a series of sensors installed around the home that can automatically track the person's movement. Using artificial intelligence to understand and predict the patterns of movement, they are then able to interpret deviations, which could indicate an event such as a fall or the onset of a urinary tract infection, as well as allowing medical professionals to monitor disease progression. The technology is still in development but deploying this kind of system within the home must involve careful and sensitive management of personal privacy and data security. In the future, supportive systems like this can allow people with dementia to continue to live in their own homes for longer, which is a profound wish for many.

Concurrently, intensive research efforts continue to identify and develop new drug treatments. Translating these promising preclinical results into interventions that benefit patients have not always been successful and have proved especially challenging in the complex field of neurodegenerative disease. Although this can be a disappointing outcome, it is also important to use these as opportunities to fully understand the reasons for any failures. As two examples published in September's issue of *EBioMedicine* show, careful analysis of the differences between humans and preclinical models and further stratification of participant cohorts can provide renewed hope for interventions with previously negative results.

The loss of cholinergic neurons is known as one of the hallmarks of Alzheimer's related neurodegeneration. As a result, alpha7 nicotinic acetylcholine receptor (α7 nAChR), a receptor that functions within the cholinergic nerve system of the brain, has been a drug target of interest for many years. Despite promising preclinical results, the drugs have failed to show success in human trials. A newly published study in *EBioMedicine* proposed that this is due to a failure to account for a unique human-specific fusion gene, *CHRFAM7A*, which is believed to function as a negative regulator of α7 nAChR. Work led by Kinga Szigeti (State University of New York, NY, USA) explored the functionality of the *CHRFAM7A* alleles using electrophysiology and Aβ_1–42_ uptake in an induced pluripotent stem cells (iPSC) model. They found that the inverted allele of *CHRFAM7A* is non-functional. This can be considered as equivalent to the background of preclinical animal models, in which *CHRFAM7A* does not exist. In two retrospective cohorts with *CHRFAM7A* genotyping, the authors then examined the response to treatment initiation and the disease modifying effect of exposure to acetylcholinesterase inhibitors (AChEI). As expected from the absence of function of the inverted *CHRFAM7A* allele in their iPSC model, an improved response to AChEI was observed in the participants who were carriers of the inversion. Furthermore, the authors provided estimates that 25% of the Alzheimer's disease population carry this non-functional allele and would therefore potentially benefit from drugs targeting α7 nAChR. This finding calls for detailed reanalysis of previous trials in which a failure to initially stratify by patient genotype means that a potential benefit of some therapies for subgroups of patients might have been missed.

Similarly, epidemiological studies have found that high levels of the omega-3 fatty acids, eicosapentaenoic acid (EPA), and docosahexaenoic acid (DHA), are associated with a lower risk of Alzheimer's disease. Promising preclinical animal work showed that long-term, high dosage supplementation with DHA reduced β-amyloid levels and decreased neuronal loss. However, in clinical trials, patients who had already been diagnosed did not benefit from DHA supplementation and symptoms continued to progress. The existing onset of cognitive impairment and the A*POE4* allele have both been proposed as factors explaining this translational gap. In a small, randomised clinical trial led by Hussein Yassine (Keck School of Medicine, CA, USA) and published in *EBioMedicine* in September researchers sought to understand the bioavailability of EPA and DHA to the brain of individuals who were cognitively normal. 33 participants, stratified by *APOE4* allele status, were randomly assigned to receive either a daily high dose (>2 g) of DHA or placebo. At 6 months, they found a significant increase in EPA and DHA levels in the cerebrospinal fluid (CSF) and plasma in participants in the supplementation groups, compared with baseline measurements within the same groups. Compared with non-carriers of *APOE4,* less of an increase in levels of DHA and EPA was seen in the carriers. Previous trials might not have used adequate dosages for brain bioavailability or accounted for allele status and so, in this way, the study can inform future trial design to ensure proper evaluation of this intervention.

WHO projects that the number of people with dementia will triple in the coming decades, up to 152 million in 2050, and therefore improving therapeutic management options is of crucial importance. *EBioMedicine* looks forward to continuing to publish high quality translational studies that support the development and exploration of novel therapeutic options for those with neurocognitive disease.

*EBioMedicine*

